# Impact of the Age-adjusted Charlson comorbidity index on the short- and long-term outcomes of patients undergoing curative gastrectomy for gastric cancer

**DOI:** 10.7150/jca.35465

**Published:** 2019-08-29

**Authors:** Yukio Maezawa, Toru Aoyama, Kazuki Kano, Hiroshi Tamagawa, Masakatsu Numata, Kentaro Hara, Masaaki Murakawa, Takanobu Yamada, Tsutomu Sato, Takashi Ogata, Takashi Oshima, Norio Yukawa, Takaki Yoshikawa, Munetaka Masuda, Yasushi Rino

**Affiliations:** 1 Department of Surgery, Yokohama City University, 3-9, Fukuura, Kanazawa-ku, Yokohama, Kanagawa 236-0004, Japan; 2 Department of Gastrointestinal Surgery, Kanagawa Cancer Center, 2-3-2, Nakao, Asahi-ku, Yokohama, Kanagawa 241-8515, Japan.; 3 Department of Gastric Surgery, National Cancer Center Hospital, 5-1-1 Tsukiji, Chuo-Ku, Tokyo 104-0045, Japan.

**Keywords:** Age-adjusted Charlson comorbidity index, comorbidity, postoperative complications, overall survival, gastric cancer

## Abstract

**Background**: The aim of this study was to determine whether or not the short- and long-term outcomes were affected by the age-adjusted Charlson comorbidity index (ACCI) in patients who underwent curative resection for gastric cancer.

**Methods**: The patients were retrospectively selected from among the medical records of consecutive patients who underwent curative gastrectomy with nodal dissection for gastric cancer at Yokohama City University and Kanagawa Cancer Center from January 2000 to August 2015.

**Results**: A total of 2254 patients were eligible for inclusion in the present study. One thousand six hundred fifty-six patients had an ACCI of <6 points (ACCI low group), while 598 had a score of ≥6 points (ACCI high group). The median age (p<0.001) and American Society of Anesthesiologists physical status (ASA-PS) score (p<0.001) of the ACCI high group were higher in comparison to the ACCI low group. The incidence of surgical complications in the ACCI high group was significantly higher than that in the ACCI low group (12.0% vs. 7.2%, p<0.001). Univariate and multivariate analyses demonstrated that an ACCI high classification was a significant risk factor for postoperative complications. In addition, the 5-year OS rates of the ACCI low and ACCI high groups were 85.4% and 74.1%, respectively. The difference was statistically significant (p<0.001). The univariate and multivariate analyses demonstrated that an ACCI high classification was a significant prognostic factor for OS.

**Conclusions**: Our results support that a high ACCI value is an independent risk factor for the short- and long-term outcomes of patients with gastric cancer. To improve the survival of patients with gastric cancer, it is necessary to carefully plan the perioperative care and the surgical strategy according to the ACCI.

## Background

A total of 951,600 new cases and 723,100 deaths due to gastric cancer occurred worldwide in 2012 1. Complete resection is essential for the cure of localized gastric cancer. However, the morbidity and mortality rates of patients who undergo radical gastrectomy for gastric cancer are reported to be 20-40% and 1-5%, respectively 2,3. Recently, the proportion of elderly patients diagnosed with gastric cancer has tended to increase 4. Elderly patients are likely to have more comorbidities and the presence of comorbidities is considered to be associated with a higher risk of morbidity and mortality 5. Several previous studies have shown that the presence of comorbidities and their severity affects both the short- and long-term outcomes after gastric cancer surgery 6. However, the optimal tools for comprehensively evaluating the various comorbid diseases in gastric cancer treatment have not been sufficiently established.

The Charlson comorbidity index (CCI), which was first proposed by Charlson et al. in 1987, has been extensively used to evaluate the impact of comorbidity in a variety of cancers and non-cancer conditions 7. The CCI is a prognostic taxonomy that is considered to be useful for prognostic prediction by weighing and scoring each comorbidity disease. It was initially developed to account for the influence of patients' adverse medical conditions on longitudinal studies. The age-adjusted Charlson comorbidity index (ACCI), corrects the final CCI score for the age of the patient. Although several studies evaluated the clinical impacts of the ACCI in the patients with various types of malignancies, most previous studies have used and evaluated the data with relatively small sample sizes of less than 200 from a single institution. Small sample sizes data have many limitations, such as unspecified indications of surgery, heterogeneous populations, heterogeneous treatments, and description bias of surgical morbidity. To overcome such limitations associated with small sample sizes data, we focused on cases that were enrolled in large individual patients' data collecting from multi institutes 8,9,10.

The aim of this study was to determine whether or not the short- and long-term outcomes of the patients in a large database who underwent curative resection for gastric cancer were affected by the ACCI. This study had the ultimate goal of evaluating and confirming the actual impact of ACCI on the treatment outcomes of gastric cancer patients who received curative surgery.

## Patients and Methods

### Patients

The patients were retrospectively selected from among the medical records of consecutive patients who underwent gastrectomy with nodal dissection for gastric cancer at Yokohama City University and Kanagawa Cancer Center from January 2000 to August 2015, according to the following criteria: (1) histologically proven gastric adenocarcinoma, (2) D2 or D1+ gastrectomy with curative lymph node dissection as the first treatment, and (3) achieved complete (R0) resection.

### Surgical procedure and pathological findings

In principle, D2 gastrectomy was selected for T2-T4 disease, whereas D1+ was selected for T1 cancer according to the Japanese gastric cancer treatment guidelines ver. 3 11. The resected specimens were histopathologically examined and staged according to the Japanese classification of gastric carcinoma: 3rd English edition 12.

### Definition of postoperative complications

Postoperative complications of grade 2-5 according to the Clavien-Dindo classification that occurred during hospitalization and/or within 30 days after surgery were retrospectively determined from the patient's records 13. Grade 1 complications were not evaluated in order to exclude the possibility of a description bias in the patient's records.

### Adjuvant treatment

All patients who underwent radical gastrectomy from January 2000 to August 2006 were followed up at an outpatient clinic and received surgery alone. In July 2006, the Adjuvant Chemotherapy Trial of S-1 for Gastric Cancer (ACTS-GC) demonstrated the efficacy of S-1 as adjuvant chemotherapy for Japanese patients undergoing D2 curative gastrectomy for locally-advanced gastric cancer (pathological stage II or III disease) 14. Based on the ACTS-GC trial, S-1 adjuvant chemotherapy became the standard treatment for patients with stages II and III gastric cancer.

### Follow-up

Patients were followed up at outpatient clinics. In principle, hematological tests and physical examinations were performed at least every three months for the first three years after surgery and then every six months until five years after surgery. The serum CEA and CA19-9 levels were checked at least every three months for five years. Patients underwent a computed tomography (CT) examination every six months during the first three years after surgery and then every twelve months until five years after surgery.

### Measurement of the age-adjusted Charlson co morbidity index (ACCI)

We used the comorbidity index developed by Charlson et al. to quantify baseline comorbidities 8. The weighted age and comorbidity values are shown in **Table 1**. Information on pre-existing comorbidities that were present before the gastrectomy were available from the medical records of Yokohama City University and Kanagawa Cancer Center. The index is a weighted measure that incorporates 19 different medical categories, each of which is weighted according to its potential impact on mortality. Conditions with a weight of one included: myocardial infarction, congestive heart failure, peripheral vascular disease, cerebrovascular disease, dementia, chronic pulmonary disease (COPD), connective tissue disease, ulcer disease, mild liver disease and diabetes mellitus without end-organ damage. Conditions with a weight of two included: hemiplegia, moderate or severe chronic kidney disease, diabetes with end-organ damage, solid tumor, leukemia and lymphoma. Moderate or severe liver disease (e.g., cirrhosis with ascites) was given a weight of 3 and metastatic solid tumor or Acquired immunodeficiency syndrome (AIDS) was given a weight of 6. The final score of each patient was calculated by taking all comorbid conditions into account. The ACCI was calculated with additional points added for age (1 point was added for each decade over 40 years of age). The basic value of “2” in the CCI was applied to all patients (because of gastric cancer). The patients were classified into the low or high ACCI groups according to their score: less than 6 (low ACCI), and 6 or more (high ACCI).

### Statistical analyses

The significance of the correlation between the ACCI and clinicopathological parameters was determined using Fisher's exact test or the χ^2^ test and the Mann-Whitney U test for continuous variables. Overall survival (OS) was defined as the period between surgery and death. Recurrence-free survival (RFS) was defined as the period between surgery and recurrence or death, whichever came first. The data of the patients who did not experience an event was censored on the date of the final observation. The OS and RFS were evaluated by univariate and multivariate analyses, and the OS and RFS curves were calculated using the Kaplan-Meier method and compared by the log-rank test. Cox's proportional hazard model was used to perform univariate and multivariate survival analyses. To select a model, we used backward elimination in the multivariate analysis. The impact of the ACCI on postoperative morbidity and mortality were examined using Fisher's exact test or the χ^2^ test. Postoperative morbidity was evaluated by univariate and multivariate analyses and a logistic regression analysis was performed. Patients with missing covariate values were excluded. Standard clinical thresholds were used, dividing the continuous variables into no more than two categories. P values of <0.05 were considered to indicate statistical significance. The survival data were obtained from hospital records or from the city registry system. The SPSS software program (ver. 23.0; IBM Corp., Armonk, NY, USA) was used to perform all of the statistical analyses. This study was approved by the Institutional Review Board (IRB) of the Yokohama City University (IRB Number: B160707003) and Kanagawa Cancer Center (2016.epidemiologic study-22).

## Results

### The background characteristics of the patients

A total of 2254 patients were eligible for inclusion in the present study. The patients' ages ranged from 24 to 87 years (median: 65 years); 1539 were male, and 715 were female. The American Society of Anesthesiologists physical status (ASA-PS) values of the patients were as follows ASA-PS 1, n= 860 (38.2%); ASA-PS 2, n=1371 (60.8%); and ASA-PS 3, n=23 (1.0%). The median follow-up period was 61 months (1-180 months). Sixty percent of the patients received distal gastrectomy, and 80% of patients received D2 lymph node dissection. One thousand six hundred fifty-six patients had an ACCI of <6 points and were classified into the ACCI low group; 598 had an ACCI of ≥6 points and were classified into the ACCI high group. **Table 2** summarizes the patients' demographic information and compares the clinical characteristics between the ACCI low and ACCI high groups. The median age (p<0.001) and ASA-PS (p<0.001) of the ACCI high group were higher than those in the ACCI low group. There were significant differences in operation time and blood loss between the two groups; however, both differences were small. There was no significant difference in the pathological stage. We also analyzed the relationship between the ACCI value and every TNM stage (7^th^ edtion), and there was no relation between the ACCI value and every TNM stage (p=0.089) 15.

### Postoperative complications

Postoperative complications were found in 191 (8.5%) of the 2254 patients. The most frequent postoperative complication was pancreatic fistula (2.8%), followed by anastomotic leakage (1.8%), anatomic stenosis (1.7%), ileus (1.3%), and surgical site infection (SSI) (1.1%). These complications were more frequent than 1.0%. Surgical mortality was observed in four patients, all of whom were in the ACCI high group. The incidence of surgical complications in the ACCI high group was significantly higher than that in the ACCI low group (12.0% vs. 7.2%, p<0.001). Infectious complications, such as pancreatic fistula, anastomotic leakage, SSI, pneumonia, abdominal abscess and catheter infection, were significantly more frequent in the ACCI high group (p=0.009). Interestingly, the incidence of pancreatic fistula in the ACCI high group was significantly higher than that in the ACCI low group. However, the incidence of pancreatic fistula was not related to somehow more extensive surgical procedure such as the extent of nodal dissection (p=0.47), surgical extension to pancreatic tail (p=0.78) and frequency of far advanced tumors (T4a/T4b or not; p=0.53). On the other hand, the incidence of non-infectious complications, such as anatomic stenosis, ileus and lymphatic fistula, did not differ between the two groups to a statistically significant extent (p=0.22). The univariate and multivariate analyses demonstrated that an ACCI value of ≥6 was a significant risk factor for postoperative complications (HR 1.66; 95 % CI 1.21-2.28, P = 0.002) (**Table 4**). The median was used for the boundaries of operation time and blood loss.

### Survival analyses

**Figure 1:**
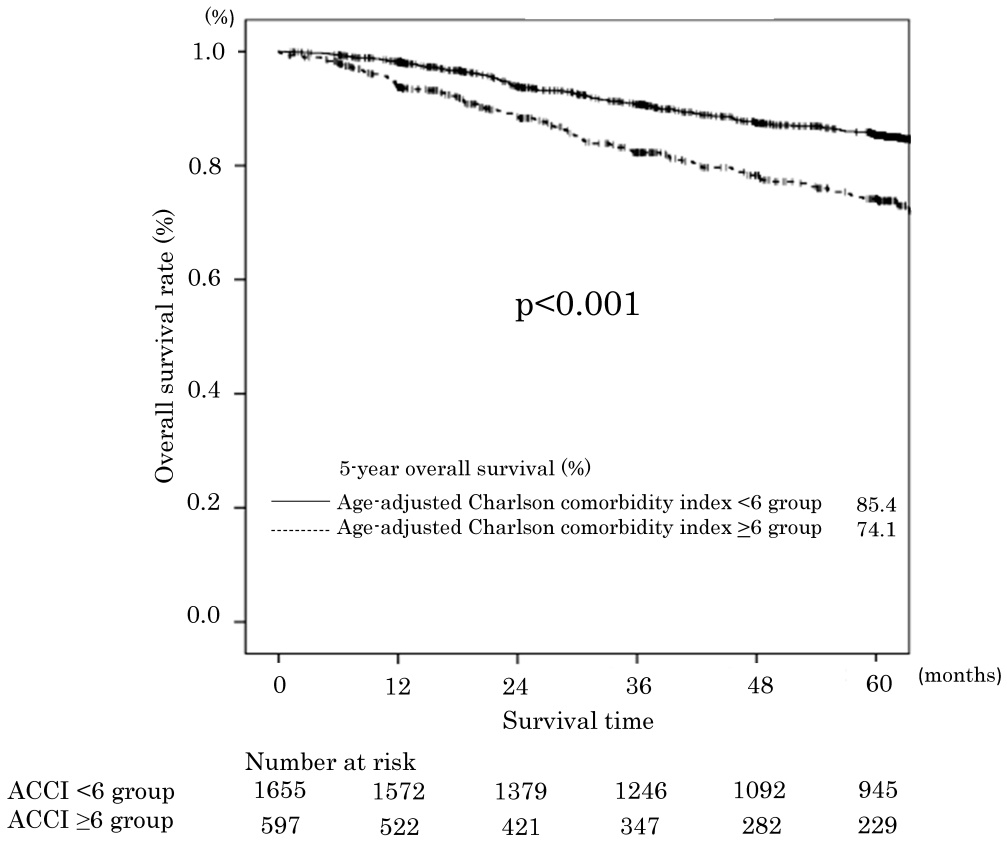
Overall survival in the age-adjusted Charlson comorbidity index <6 and ≥6 groups.

The median follow-up time for the 2254 patients was 60 (range: 0-177) months. The 5-year OS rates of the ACCI low and ACCI high groups were 85.4% and 74.1%, respectively. The difference was statistically significant (p<0.001). The OS curves are shown in **Figure 1**. Two hundred thirty-eight 0f 406 patients died of gastric cancer within this period. The OS was significantly worse in the ACCI high group, with an unadjusted hazard ratio (HR) for complications of 1.73 (95 %confidence interval [CI] 1.40-2.13; P<0.0001). Before multivariate analysis, T and N factor (instead of pStage) were excluded to alleviate the multicollinearity. The univariate and multivariate analyses demonstrated that an ACCI value of ≥6 was a significant prognostic factor for OS (HR 1.80; 95 % CI 1.46-2.23, P < 0.001). The ASA-PS was not a significant prognostic factor (**Table 4**).

**Figure 2:**
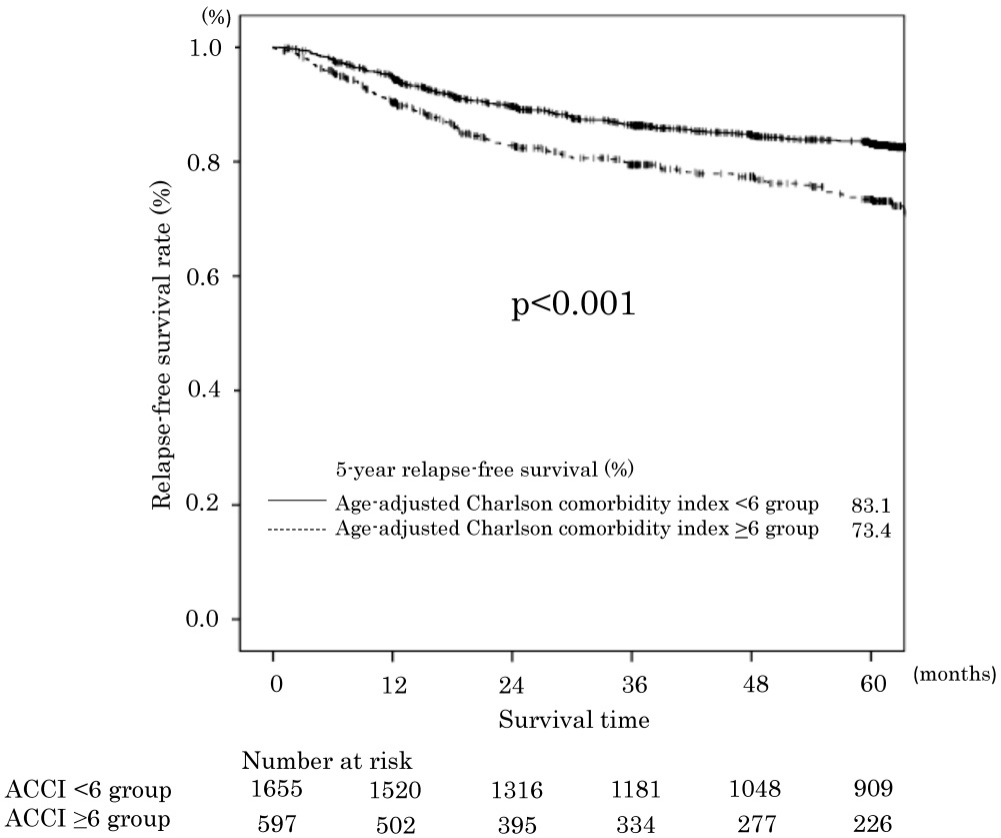
Relapse-free survival in the age-adjusted Charlson comorbidity index <6 and ≥6 groups.

The 5-year RFS rates in the ACCI low and ACCI high groups were 83.1% and 73.4%, respectively. The difference was statistically significant (p<0.001). The RFS curves are shown in **Figure 2**. The univariate and multivariate analyses also demonstrated an ACCI value of ≥6 was a significant prognostic factor for RFS (HR 1.44; 95 % CI 1.15-1.80, P = 0.001). The 5-year cancer specific overall survival (CSS) in the ACCI low and ACCI high groups were 89.1% and 85.7%, respectively. The difference was statistically significant (p=0.016).

## Discussion

The present study aimed to determine whether or not the ACCI had a clinical impact on gastric cancer patients who underwent curative gastrectomy. The major finding of the present study was that both the short- and long-term outcomes of gastric cancer patients were affected by the ACCI. Our results support that a high ACCI was an independent risk factor for postoperative complications and poorer OS. To improve the survival of gastric cancer patients, it is necessary to carefully plan the surgical procedure, perioperative care and the surgical strategy using the ACCI.

The present study demonstrated that the ACCI was an independent risk factor for surgical complications in patients who underwent curative surgery for gastric cancer. The hazard ratio for surgical complications was 1.66 (95% confidence interval, 1.21 to 2.28). Similar results have been observed in other malignancies. Kahl et al. analyzed the prognostic impact of the ACCI on both postoperative morbidity and overall survival (OS) in 793 patients with advanced epithelial ovarian cancer 16. The patients were classified into low ACCI (0-1), intermediate ACCI (2-3), and high ACCI (>4) groups. They demonstrated that intermediate and high ACCI values were significantly associated with severe postoperative complications, defined as Clavien-Dindo classification grade 3-5 (Intermediate ACCI: OR 1.55, 95% CI 1.06-2.26, p=0.026, High ACCI: OR 3.27, 95% CI 1.97-5.43, p<0.001). Other reports showed similar results 9, 17. In addition, the incidence of infectious complications in the ACCI high group was significantly higher than that in the ACCI low group (p=0.001), while the incidence of noninfectious complications did not differ to a statistically significant extent. Thus, careful attention should be paid to the possible development of surgical complications, especially infectious complications, in patients with high ACCI values when surgeons perform curative gastrectomy for gastric cancer.

The present study also demonstrated that the ACCI was an independent risk factor for OS in the patients who underwent curative surgery for gastric cancer. There are several possible reasons why the ACCI might have affected the long-term outcomes of gastric cancer patients. One possible reason is that the ACCI might be associated with postoperative surgical complications. As mentioned above, the incidence of postoperative infectious complications in the ACCI high group was higher than that in the ACCI low group in the present study. Recent studies have demonstrated that the development of postoperative complications is associated with decreased survival or an increased risk of disease recurrence in various types of malignancies 18-26. Actually, we previously investigated the impact of postoperative complications on gastric cancer survival and recurrence after curative surgery 27. Another possible reason for this association is that the patients who were in the ACCI high group might have had some factors that led to decreased host immunity against their tumors. For example, Goldfarb et al. reported that treatment aimed at the perioperative enhancement of cell-mediated immunity with the simultaneous inhibition of excessive catecholamine and prostaglandin responses could be successful in limiting postoperative immune suppression and metastatic progression 28. In addition, Dunn et al. suggested that the adaptive immune system could function by identifying and eliminating nascent tumor cells in experimental models 29. However, these mechanisms were speculative, and further work is clearly needed to investigate them.

Present study also demonstrated the relation of ACCI and postoperative complications rate. Pancreatic fistula occurred in the most patients and the incidence was 2.8% in all patients. Furthermore, present study demonstrated the significantly high incidence of pancreatic fistula in the ACCI high group (4.3%) than that in the ACCI low group (2.3%) was shown and the higher pancreatic fistula rate in the ACCI high group was indeed related to comorbidity and not to a somehow more extensive surgical procedure. However, we used the fistula grading according to Clavien- Dindo (not the Bassi classification). Therefore, the frequency of pancreatic fistula might be underestimated.

Although the present results are considered to be solid in terms of the follow-up period and sample size, the present study is associated with several limitations. First, the present study was retrospective in nature, and might have contained a selection bias. Second, there was a time bias in this study, as the data were collected over a relatively long period (2000-2015), the surgical procedures, perioperative care, and adjuvant chemotherapy might have changed over the years 30, 11. Third, the definition and severity of morbidities were not strictly defined in this study. The postoperative surgical complications were reported based on the judgment of individual physicians rather than the study protocol. Although the incidence of morbidity in this study was similar to that in other large studies, the incidence of some surgical complications might have been underestimated 31, 32. A further important limitation of all of the available data regarding ACCI, including the date used in the current study, is the lack of consensus regarding the most appropriate cut-off for the evaluation of the ACCI 10, 16, 33, 34. In our study, we used a cut-off value of 6 according to previous reports that evaluated all endpoints. However, there needs to be a consensus regarding the definitions of 'high' versus 'low' ACCI. This would also greatly aid the use of the ACCI as a stratification factor in future studies.

## Conclusion

The short- and long-term outcomes of gastric cancer patients were affected by the ACCI. Our results support that a high ACCI was an independent risk factor for postoperative complications and poorer OS. To improve the survival of gastric cancer patients, it is necessary to carefully plan the perioperative care and the surgical strategies using the ACCI in daily clinical practice.

## Figures and Tables

**Table 1:** Weighted index of comorbidities in Charlson comorbidity index

Comorbidities	Assigned weights for comorbidities
Myocardial infarction	1
Congestive heart failure	1
Peripheral vascular disease	1
Cerebrovascular disease	1
Dementia	1
Chronic obstructive pulmonary disease (COPD)	1
Connective tissue disease	1
Ulcer disease	1
Mild liver disease	1
Diabetes mellitus without end‐organ damage	1
Hemiplegia	2
Moderate to severe chronic kidney disease	2
Diabetes with end‐organ damage	2
Solid tumor	2
Leukemia	2
Lymphoma	2
Moderate to severe liver disease	3
Metastatic solid tumor	6
Acquired immunodeficiency syndrome (AIDS)	6

Add 1 point per decade to ages over 40 years.

**Table 2:** Comparison of patient background factors between patients with an age-adjusted Charlson comorbidity index value of <6 and those with an age-adjusted Charlson comorbidity Index value of ≥6

Variables	All patients (n=2254)	ACCI<6 group (n=1656)	ACCI≥6 group (n=598)	*p* value
Age (years), median (range)	65 (24-87)	62 (24-79)	74 (60-87)	<0.001
Gender				0.008
Female	715 (31.7%)	551 (33.3%)	164 (27.4%)	
Male	1539 (68.3%)	1105 (66.7%)	434 (72.6%)	
Body mass index, median (range)	22.3 (13.7-39.5)	22.2 (13.7-39.5)	22.7 (14.8-33.0)	0.001
ASA-PS				<0.001
1	860 (38.2%)	831 (50.2%)	29 (4.8%)	
2	1371 (60.8%)	819 (49.5%)	552 (92.3%)	
3	23 (1.0%)	6 (0.4%)	17 (2.8%)	
Surgical procedure				0.140
Total	793 (35.2%)	568 (34.3%)	225 (37.6%)	
Distal	1390 (61.7%)	1040 (62.8%)	350 (58.5%)	
Others	71 (3.1%)	48 (2.9%)	23 (3.8%)	
Nodal dissection				<0.001
D1+	482 (21.4%)	315 (19.0%)	167 (27.9%)	
D2	1772 (78.6%)	1341 (81.0%)	431 (71.1%)	
Operation time (min), median (range)	190 (68-731)	190 (68-731)	195 (71-609)	0.005
Blood loss (ml), median (range)	135 (5-2510)	130 (5-2510)	150 (5-2230)	0.006
Pathological Stage				0.540
I	1440 (63.9%)	1067 (64.4%)	373 (62.4%)	
II	395 (17.5%)	290 (17.5%)	105 (17.6%)	
III	419 (18.6%)	299 (18.1%)	120 (20.1%)	
Postoperative surgical complication				0.009
No	2079 (92.2%)	1542 (93.1%)	537 (89.8%)	
Yes	175 (7.8%)	114 (6.9%)	61 (10.2%)	
Adjuvant chemohetrapy				0.750
No	1823 (80.9%)	1342 (81.0%)	481 (80.4%)	
Yes	431 (19.1%)	314 (19.0%)	117 (19.6%)	
Absolute CCI values, median (range)	2 (2-4)	2 (2-4)	3 (2-4)	<0.001
pT stages				0.001
1	1313 (58.3%)	975 (58.8%)	338 (56.5%)	
2	318 (14.1%)	228 (13.8%)	90 (15.1%)	
3	197 (8.7%)	133 (8.0%)	64 (10.7%)	
4	426 (18.9%)	320 (19.4%)	106 (17.7%)	
pN stages				0.306
0	1530 (67.9%)	1113 (68.4%)	397 (66.4%)	
1	279 (12.4%)	202 (12.2%)	77 (12.9%)	
2	204 (9.1%)	138 (8.3%)	66 (11.0%)	
3	241 (10.7%)	183 (11.1%)	58 (2.5%)	
Number of dissected lymph nodes, median (range)	45 (0-184)	46 (0-184)	42 (3-139)	0.001
Frequency of lymphatic vessel infiltration				0.090
No	1606 (71.3%)	1196 (72.2%)	410 (68.6%)	
Yes	648 (28.7%)	460 (27.8%)	188 (31.4%)	
Lauren histological type				<0.001
Intestinal	1032 (45.8%)	704 (42.5%)	328 (54.8%)	
Diffuse	1222 (54.2%)	952 (57.5%)	270 (45.2%)	
Tumor size (mm), median (range)	37 (0-212)	35.5 (0-212)	38 (0-210)	0.142
Number of minimal invasive procedures				0.621
Open	1683 (74.7%)	1241 (74.9%)	442 (73.9%)	
Laparoscopic	571 (25.3%)	415 (25.1%)	156 (26.1%)	
Length of hospital stay (in days), median (range)	10 (2-172)	10 (6-137)	10 (2-172)	0.844
Surgical extension to other organs				0.610
No	2227 (98.8%)	1635 (98.7%)	592 (99.0%)	
Yes	27(1.2%)	21 (1.3%)	6 (1.0%)	
Frequency of redo surgery				0.136
No	2210 (98.0%)	1628 (98.3%)	582 (97.3%)	
Yes	44 (2.0%)	28 (1.7%)	16 (2.7%)	
Distribution of the respective Clavien Dindo stages				<0.001
0-I	1957 (86.8%)	1470 (88.8%)	487 (81.4%)	
II	194 (8.6%)	122 (7.4%)	72 (12.0%)	
IIIa	55 (2.4%)	36 (2.2%)	19 (3.2%)	
IIIb	35 (1.5%)	22 (1.3%)	13 (2.2%)	
IVa	8 (0.4%)	5 (0.3%)	3 (0.5%)	
IVb	1 (0.04%)	1 (0.1%)	0 (0.0%)	
V	4 (0.2%)	0 (0.0%)	4 (0.7%)	

*ASA-PS* American Society of Anesthesiologists physical status, *CCI* Charlson comorbidity index, *ACCI* Age-adjusted Charlson comorbidity index

**Table 3:** Univariate and multivariate logistic regression analyses of the clinicopathological factors associated with postoperative complications

Characteristics	Number	Univariate			Multivariate		
		HR	95%CI	P value	HR	95%CI	P value
Age (years)				0.016			
< 65	1038	1.00					
≥ 65	1216	1.46	1.07-1.98				
Gender				<0.001			0.002
Female	715	1.00			1.00		
Male	1539	2.04	1.41-2.95		1.84	1.25-2.71	
Body mass index				0.007			
< 25	1831	1.00					
≥ 25	423	1.61	1.14-2.26				
Operation time				<0.001			<0.001
< 190 min	1131	1.00			1.00		
≥ 190 min	1123	2.13	1.56-2.91		2.00	1.45-2.76	
Blood loss				0.017			0.003
< 140 ml	1131	1.00			1.00		
≥ 140 ml	1123	1.92	1.41-2.62		1.62	1.17-2.22	
Nodal dissection				0.44			
D2	1772	1.00					
D1+	482	1.15	0.81-1.63				
ASA-PS				0.002			
1	860	1.00					
2-3	1394	1.67	1.20-2.32				
ACCI				<0.001			0.002
< 6	1656	1.00			1.00		
≥ 6	598	1.77	1.30-2.41		1.66	1.21-2.28	

*ASA-PS* American Society of Anesthesiologists physical status, *ACCI* Age-adjusted Charlson comorbidity index

**Table 4:** Univariate and multivariate Cox proportional hazards analyses of the clinicopathological factors associated with overall survival

Characteristics	Number	Univariate			Multivariate		
		HR	95%CI	P value	HR	95%CI	P value
Age(years)				<0.001			
< 65	1038	1.00					
≥ 65	1216	1.47	1.21-1.80				
Gender				0.048			0.020
Female	715	1.00			1.00		
Male	1539	1.25	1.00-1.56		1.32	1.04-1.66	
Pathological stage				<0.001			<0.001
I	1440	1.00			1.00		
II	395	3.07	2.34-4.03		2.84	2.16-3.73	
III	419	7.55	6.00-9.51		6.02	4.73-7.66	
Infectious complication				0.015			0.012
No	2079	1.00			1.00		
Yes	175	1.50	1.08-2.07		1.52	1.10-2.11	
ASA-PS				<0.001			
1	860	1.00					
2-3	1394	1.51	1.23-1.86				
ACCI§				<0.001			<0.001
< 6	1656	1.00			1.00		
≥ 6	598	1.73	1.40-2.13		1.80	1.46-2.23	

*ASA-PS* American Society of Anesthesiologists physical status, *ACCI* Age-adjusted Charlson comorbidity index

## Abbreviations

ACCI: age-adjusted Charlson comorbidity index
ACTS-GC: Adjuvant Chemotherapy Trial of S-1 for Gastric Cancer
AIDS: Acquired immunodeficiency syndrome
ASA-PS: American Society of Anesthesiologists physical status
CA19-9: carbohydrate antigen 19-9
CCI: Charlson comorbidity index
CEA: Carcinoembryonic antigen
CI: Confidence interval
COPD: Chronic obstructive pulmonary disease
CT: computed tomography
OR: Odds ratio
OS: Overall survival
RFS: Recurrence-free survival
SSI: surgical site infection
